# Effects of Replacing Soybean Meal with Cottonseed Meal, Peanut Meal, Rapeseed Meal, or Distillers’ Dried Grains with Solubles on the Growth Performance, Nutrient Digestibility, Serum Parameters, and Rumen Fermentation in Growing Lambs

**DOI:** 10.3390/vetsci11070322

**Published:** 2024-07-17

**Authors:** Xuejiao Yin, Meijing Chen, Caihong Yang, Chunhui Duan, Shoukun Ji, Hui Yan, Yueqin Liu, Yingjie Zhang

**Affiliations:** 1 College of Animal Science and Technology, Hebei Agricultural University, Baoding 071000, China; bdyinxuejiao@foxmail.com (X.Y.); chenmeijing815@126.com (M.C.); yangcaihong66@163.com (C.Y.); duanchh211@126.com (C.D.); jishoukun@163.com (S.J.); yanhuihui@126.com (H.Y.); 2 College of Animal Science and Technology, Hebei Normal University of Science and Technology, Qinhuangdao 066004, China; 3 Key Laboratory of Specialty Animal Germplasm Resources Exploration and Innovation, Qinhuangdao 066004, China

**Keywords:** protein source, growing lambs, rumen fermentation, feed intake, feed efficiency

## Abstract

**Simple Summary:**

Intensively managing the use of concentrate supplementation to complement nutrition can increase production costs and reduce producers’ income. Considering the frequently large price fluctuations for soybean meal, an alternative is the replacement of local protein sources in ruminant feeding. In this study, we compared the effects of different sources of protein (soybean meal, cottonseed meal, peanut meal, rapeseed meal, and distillers’ dried grains with solubles) on the growth, digestibility, and rumen fermentation of growing lambs. Our results showed that when soybean meal was totally replaced with either cottonseed meal, peanut meal, rapeseed meal, or distillers’ dried grains with solubles, there was no impact on the average daily gain of growing lambs, but digestibility was reduced. Our study provides a theoretical basis for the rational selection and utilization of proteins from different sources, which helps us to optimize the feeding management of growing lambs.

**Abstract:**

Considering the frequently large price fluctuations for soybean meal, an alternative is the increased use of locally produced high-protein ingredients. The objective of this study was to evaluate the effects of the total replacement of soybean meal with different sources of protein on the growth performance, nutrient digestibility, serum parameters, rumen fermentation parameters, and bacterial communities in growing lambs. Sixty sheep with similar body weights (38.46 ± 0.71 kg) were distributed to one of five treatments: soybean meal (SBM); cottonseed meal (COM); peanut meal (PEM); rapeseed meal (RAM); and distillers’ dried grains with solubles (DDGS). The experiment lasted 62 days with a 10-day adaptation period and a 52-day growing period. The results indicated that the body weight and average daily gain were not affected by different protein sources (*p* > 0.05), but the dry matter intake of the SBM group was lower than that of the other groups (*p* < 0.05); otherwise, the feed efficiency was higher (*p* < 0.05). The digestion of dry matter was higher in the SBM, COM, and RAM groups than in the DDGS and PEM groups (*p* < 0.05). Meanwhile, compared to the other groups, the SBM group had the highest digestion of gross energy and crude protein (*p* < 0.05). In addition, the concentration of glutathione peroxidase was highest in the SBM group (*p* < 0.05). Regarding the rumen fermentation, the SBM group had the highest concentration of NH_3_-N (*p* < 0.05). The rumen bacterial community was not affected by treatments (*p* > 0.05). In conclusion, the total replacement of soybean meal with cottonseed, peanut, rapeseed, or DDGS reduced digestibility but did not impact the body weight or average daily gain of growing lambs and had no effect on the immune function and rumen bacterial community; thus, they can be used to substitute the soybean meal.

## 1. Introduction

Dietary protein source has significant relevance due to its direct impact on ruminant performance, as well as feed cost [1]. While dietary protein is vital for the maintenance of metabolic processes and production system productivity, it is an expensive nutrient in ruminant diets. The development and optimization of diversified protein sources for feed have become crucial and persistent requirements for ensuring a socially and environmentally sustainable future for the ruminant industry.

Soybean meal is a major protein source commonly used in diets for ruminants, with few options that can completely replace it and match the animals’ response. However, the availability of soybean meal is limited to the global animal industry, and the quantity produced cannot meet the requirements of animal husbandry; thus, the amount of soybean being imported is increasing quickly, especially in China [2,3]. The price of soybean meal is unstable, influencing the cost of feed for ruminants. Therefore, the replacement of soybean meal with other sources of protein in animal feed has become a long-term goal.

Apart from soybean meal, cottonseed meal, peanut meal, rapeseed meal, and distillers’ dried grains with solubles (DDGS) are all commonly used as dietary protein sources. It has been reported that cottonseed can increase the milk fat content and yield of dairy cows [4], while peanut meal can reduce the cost of feed without affecting feed efficiency and the nutritional quality indicators of the milk [5]. Rapeseed meal is a by-product of rapeseed oil production, is abundant in sulfur-containing amino acids, and has an excellent balance of essential amino acids [6]. Meanwhile, a meta-analysis showed that feeding DDGS can improve the growth performance, nutrient digestibility, and carcass weight of sheep [7,8]. Thus, these protein sources can be included in the diet and may reduce feeding costs while maintaining productivity, product quality, and well-balanced diets that meet nutritional requirements.

However, protein ingredient availability, as well as the possible effects of the total replacement of soybean meal on animal growth performance, digestibility, rumen function, and overall health, must be assessed. Previous studies on soybean meal replacement have mainly focused on dairy cows [4], growing pigs [9], and calves [10]; there has been little research using cottonseed meal, peanut meal, rapeseed meal, and DDGS as alternative sources to soybean meal to verify the performance of growing lambs. Thus, the present study was conducted to compare, comprehensively, the effects of these common dietary protein sources on the growth performance, nutrient digestibility, serum parameters, rumen fermentation, and ruminal bacterial communities of growing lambs. We wanted to test the hypothesis that one of these protein sources can replace soybean meal in balanced diets.

## 2. Materials and Methods

### 2.1. Experimental Animals and Diets

This study was approved and conducted by the Animal Care and Use Committee of Hebei Agricultural University (approval number: C2020204181). No national authority permit was required for blood collection in live animals.

This study was carried out between September 2021 and January 2022 at a sheep farm in Hengshui, China. In this study, 60 male growing lambs (Hu sheep breed, BW = 38.46 ± 0.71 kg) were selected and randomly allocated to one of five different protein source groups: soybean meal (SBM, *n* = 12); cottonseed meal (COM, *n* = 12); peanut meal (PEM, *n* = 12); rapeseed meal (RAM, *n* = 12); and distillers’ dried grains with solubles (DDG, *n* = 12). All growing lambs were kept in individual pens within groups.

Except for soybean meal, all of the protein materials in this study were purchased from Xinao Animal Co., Ltd. (Shijiazhuang, China). The soybean meal and premix were purchased from the Zhihao sheep farm (Hengshui, China). The diets were formulated to be isonitrogenous and isocaloric and to support the nutritional requirements of lamb (NRC, 2007). All growing lambs had free access to fresh water and were fed twice daily (0700 and 1500) with a total mixed ration (TMR) with approximately 5% feed refusal, as shown in Table 1. The duration of the experiment was 62 days, with a 10-day adaptation period on the experimental diets and a 52-day feeding period. The growing lambs were weighed on day 1 and day 52 before the morning feeding to calculate the average daily gain (ADG). The daily dry matter intake was determined by weighing the daily feed supply and orts for each lamb.

### 2.2. Digestibility Trial

At the end of the feeding period, a digestibility trial was conducted for 4 days on daily collected feed, feed residuals, and fecal samples before the morning feeding and stored at −20 °C. All growing lambs were used in the digestibility trial; the fecal samples were taken from the rectum of each sheep. We use the acid-insoluble ash as an internal fecal marker to determine the nutrient’s apparent digestibility [11]. From each sheep, 100 g of the fresh fecal samples were collected, and 10 mL sulfuric acid (10%) was added to prevent ammonia-N volatilization for further protein content analysis. All the samples were dried at 65 °C for 72 h, weighed, and ground to pass through a 1 mm screen for chemical analysis. The metabolizable energy was calculated as follows:

Metabolizable energy = digestible energy × 0.82.

### 2.3. Blood and Rumen Sample Collection

Six sheep were randomly chosen from each experimental group for the sample collection. Blood samples (5 mL) were collected from lambs via the jugular venipuncture in coagulation-promoting tubes on day 52 of the feeding period prior to the morning feeding. The samples were centrifuged at 3000× *g* for 20 min to collected serum and then separated into three aliquots and stored at −20 °C for further analysis.

Approximately 50 mL of rumen fluid was obtained via an oral stomach tube before morning feeding on day 52 of the feeding period [12]. The oral stomach tube was thoroughly cleaned with fresh warm water between sample collections to prevent cross-contamination between samples. The ruminal content was immediately filtered through 4 layers of sterile cotton gauze to obtain the filtrate of rumen fluid. The pH of the rumen fluid was immediately detected using a portable pH meter (Sartorius PB-10, Göttingen, German). All samples were immediately kept in liquid N and then stored at −80 °C for further analysis.

### 2.4. Laboratory Analysis

Samples of feed, feed residuals, and fecal samples were determined in duplicate for gross energy using a bomb calorimeter (Model 6300; Parr Instruments, Moline, IL, USA); dry matter (DM, method 934.01), crude protein (CP, method 968.06), ether extract (EE, method 973.18), calcium (method 935.13), and phosphorus (method 965.17) were determined according to AOAC [13]. The content of neutral detergent fiber (NDF) was determined using a method consistent with Van Soest et al. [14], with heat-stable α-amylase and sodium sulfite used in the NDF procedure and expressed inclusive of residual ash. Then, acid detergent fiber (ADF) was analyzed with a fiber analyzer (Ankom A200; Ankom Technology, Macedon, NY, USA).

The cryopreserved filtrate was thawed at 4 °C and thoroughly mixed by vortexing. Then, 10 mL of rumen fluid was taken and centrifuged at 3000× *g* for 10 min at 4 °C. Later, 5 mL of filtrate was mixed with 1 mL of metaphosphoric acid solution (25% *w*/*v*) with the internal standard 2-ethylbutyric (2 g/L) for volatile fatty acid (VFA) determination and then filtered through a 0.45 μm syringe filter (PES; Sangon biotech Co., Shanghai, China). The concentration of VFA was separated and determined via gas chromatography (Varian 450, Agilent Technologies China, Co., Ltd., Beijing, China) using a 30 m × 320 μm × 0.5 μm fused silica column (HP-INNOwax). The injector and detector temperatures were set at 220 °C and 250 °C, respectively. The column temperature was increased from 120 °C to 180 °C at 10 °C/min and held for 5 min. The concentration of ammonia-N was determined spectrophotometrically using a colorimetric method [15]. The microbial crude protein (MCP) concentration was determined using the Coomassie brilliant blue method [16,17].

All blood parameters were analyzed with commercial kits according to the manufacturer’s instructions (Nanjing JianCheng Bioengineering Institute, Nanjing, China). Serum glucose (GLU—glucose assay kit), total protein (TP—total protein quantitative assay kit), albumin (ALB—albumin assay kit), globulin (GLB—globulin assay kit), blood urea N (BUN—urea assay kit), total cholesterol (TCH—total cholesterol assay kit), glutamic oxaloacetic transaminase (AST—aspartate aminotransferase assay kit), and glutamic–pyruvic transaminase (ALT—alanine aminotransferase assay kit) were measured using automatic biochemical analyses (SYNCHRON CX5 PRO, Beckman Coulter, Fullerton, CA, USA). The absorbance (OD) was measured at 550 nm, 593 nm, and 412 nm, respectively, and concentrations were calculated based on a standard curve generated for each parameter. Based on these standard curves, the concentrations of each sample and parameter were calculated. For immune response, immunoglobulins (IgG, IgA, and IgM—immunoglobulin G assay kit, immunoglobulin A assay kit, and immunoglobulin M assay kit) were measured using ELISA test kits according to the manufacturer’s instructions, and the OD was measured at 450 nm.

### 2.5. DNA Extraction and Sequencing

The bacterial diversity in the rumen was detected using 16S rDNA high-throughput sequencing. The DNA was extracted from the rumen fluid using a TGuide S96 Magnetic Soil/Stool DNA Kit (Tiangen Biotech Co., Beijing, China) according to the manufacturer’s protocol. Subsequently, the Qubit dsDNA HS Assay Kit, Qubit 4.0 Fluorometer (Invitrogen, Thermo Fisher Scientific, Oregon, USA), and 1.8% agarose gel electrophoresis were used to determine the concentration and integrity of the DNA sample, respectively. The V3-V4 region of the bacterial 16S rRNA genes was amplified from the extracted DNA using the custom barcoded primers 338F (5′-ACTCCTACGGGAGGCAGCA-3′) and 806R (5′-GGACTACHVGGGTWTCTAAT-3′). Both the forward and reverse 16S primers were tailed with sample-specific Illumina index sequences to allow for deep sequencing. The PCR was performed in 10 µL reaction volumes containing 5 µL KOD FX Neo Buffer, 2 µL dNTP (2 mM each), 0.3 µL forward primer (10 µM), 0.3 µL reverse primer (10 µM), 0.2 µL KOD FX Neo, 50 ng of template DNA, and ddH_2_O up to 10 µL. The parameters of the PCR were under the following conditions: 95 °C for 5 min, followed by 25 cycles of 95 °C for 30 s, 50 °C for 30 s, and 72 °C for 40 s, along with an extension at 72 °C for 7 min and storage at 4 °C. Then, the PCR products were purified with Agencourt AMPure XP Beads (Beckman Coulter, Indianapolis, IN, USA) and quantified using the Qubit dsDNA HS Assay Kit and Qubit 4.0 Fluorometer (Invitrogen). After the individual quantification step, the amplicons were pooled in equal amounts. Finally, high-throughput sequencing was performed using the Illumina novaseq 6000 platform (Illumina, Santiago, CA, USA) to construct the library according to the manufacturer’s instructions. 

### 2.6. Bioinformatics Analysis

The bioinformatics analysis in this study was conducted with the aid of the BMK Cloud (Biomarker Technologies Co., Ltd., Beijing, China). Trimmomatic (version 0.33) was used to filter the raw data according to the quality of the single nucleotide [18]. Cutadapt (version 1.9.1) was used to identify and remove the primer sequences [19]. PE reads obtained from previous steps were assembled using USEARCH (version 10) [20] and followed by chimera removal through UCHIME (version 8.1) [21]. The qualified reads were analyzed with QIIME2 [22] and clustered into operational taxonomic units at a similarity level of 97% via the UPARSE Pipeline method [18]. Richness estimates and diversity indices, including Chao1 and Shannon’s index, were calculated using QIIME2 [23]. Non-metric multidimensional scaling (NMDS) based on the Bray–Curtis distances was conducted to compare all samples [24].

### 2.7. Statistical Analysis

Individual lambs were the experimental units. The parameters of growth performance, digestibility performance, serum parameters, and rumen fermentations were evaluated using one-way ANOVA through SPSS (version 21.0) after checking the independence, normality, and homogeneity. The statistical model was as follows:

*Y_i_* = *μ* + *X_i_* + *e_i_*,

where *Y_i_* is the dependent variable, μ is the overall mean, *X_i_* is the fixed effect of dietary treatments (5 groups), and *e_i_* is the residual error. When significant effects were detected between treatments, the means were compared using Duncan’s multiple range test. The composition of the bacterial community was compared using an ANOSIM test with the vegan package in R software (4.3.2). All data are shown as mean ± SEM, and significance was declared at *p* < 0.05.

## 3. Results

### 3.1. Growth Performance and Apparent Nutrient Digestibility

The growth performance results are shown in Table 2. Although the initial and final live weights did not differ among the dietary treatments, feed intake was the lowest (*p* < 0.05) in the SBM group. The sheep fed soybean meal as the protein source had the highest (*p* < 0.05) feed efficiency, and the sheep fed peanut meal had the lowest (*p* < 0.05).

The nutrient digestibility results are presented in Table 2. Compared to the SBM group, the digestibility of DM was lower (*p* < 0.05) in the PEM and the DDG groups. The digestibility of gross energy and crude protein was highest (*p* < 0.05) in the SBM group compared to the other groups. The EE, NDF, and ADF apparent digestibility were similar (*p* > 0.05) among all of the protein dietary treatments. 

### 3.2. Serum Parameters

The serum parameters of the different protein source diets are shown in Table 3. The RAM group had the lowest (*p* < 0.05) level of IgG, and the COM group had a lower level (*p* < 0.05) IgM. Compared to the SBM group, the sheep of the RAM group had lower (*p* < 0.05) TP, ALB, and GLB concentrations in the serum. The concentrations of the BUN in the serum of the PEM and RAM groups were lower (*p* < 0.05) than those of the other dietary groups. Meanwhile, the serum level of AST was lower (*p* < 0.05) in the PEM group than the COM group, and the serum level of ALT was highest (*p* < 0.05) in the COM group. The serum concentrations of T-AOC, MDA, IgA, GLU, and TCH were not affected by the treatment (*p* > 0.05).

### 3.3. Rumen Fermentation Parameters

Table 4 shows that the concentration of rumen ammonia-N was highest (*p* < 0.05) in the SBM group compared to the other groups. Compared to the SBM group, the content of MCP was lower (*p* < 0.05) in the DDG group. However, feeding different experimental diets did not (*p* > 0.05) affect rumen pH, total volatile fatty acids production, and acetate, propionate, butyrate, valerate, isobutyrate, and isovalerate content.

### 3.4. Changes in Rumen Microbiota and Taxonomic Composition

**Figure 1:**
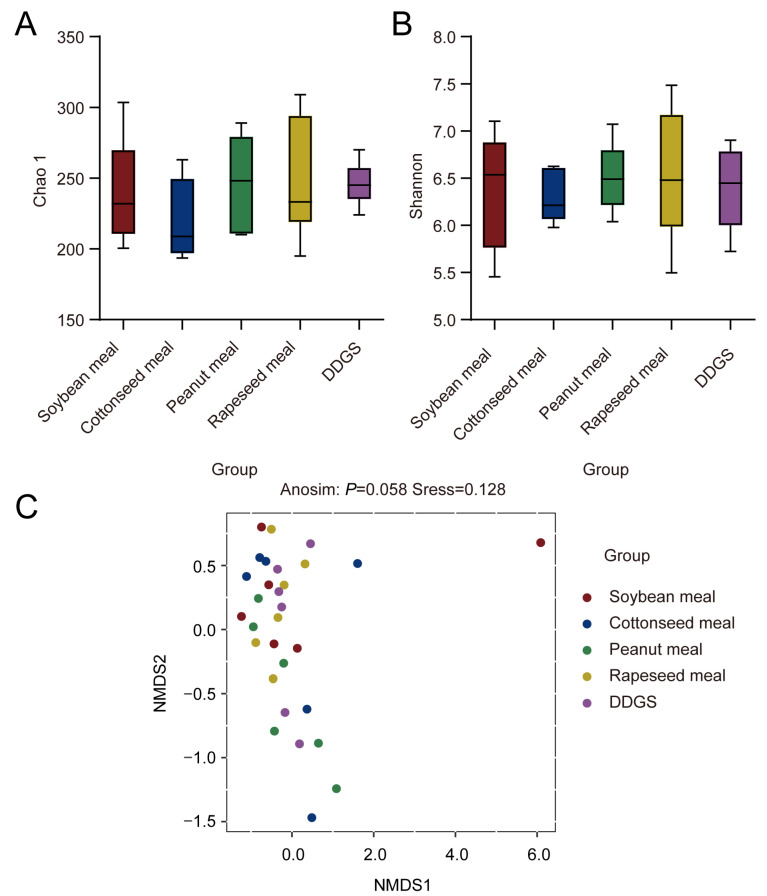
Effects of different protein sourced diets on rumen bacterial diversity: (**A**) Chao 1 index; (**B**) Shannon index; and (**C**) principal coordinate analysis (PCoA) of Bray–Curtis distance.

We compared the bacterial communities among the different protein diets, as shown in Figure 1. The alpha (Figure 1A,B) and beta diversities (Figure 1C) were not affected (*p* > 0.05) by the different diets. By focusing on the dominant bacteria with a relative abundance > 1%, we identified four dominant phyla and 10 dominant genera in the rumen, which all remained unaffected (Table 5 and Table 6, *p* > 0.05) by the different dietary treatments. A high Firmicutes/Bacteroidetes ratio (2.27) was recorded in the COM group, followed by PEM (1.98), DDG (1.56), SBM (1.22), and RAM (1.17); however, these changes were not significant (*p* > 0.05) among the five diets (Table 5).

## 4. Discussion

### 4.1. Growth Performance and Nutrient Digestibility

There has been a growing interest in further studying the sources of true protein used in ruminant feeding which play a vital role in the health and productivity of animals [25]. In the present study, we found that the dietary protein source did not affect the live weight or ADG of the growing lambs, but decreased ADFI and increased feed efficiency were observed in the SBM group. Compared to other dietary protein sources, the rumen degradation rate of soybean meal may be faster, as reported by Yang et al. [26]. This suggests that sheep fed SBM can degrade in a relatively short period of time, thus signaling the feedback center and causing decreased appetite, reduced feed intake, and increased feed efficiency [27]. In addition, the lower ADFI observed in the SBM group leads to higher apparent digestibility in the GE and CP through the increased residence time in the rumen. A previous study in which COM completely replaced either SBM or expeller SBM (ESM) demonstrated that in lactating dairy cows, the total tract digestible protein level was lower for the COM diet compared with soybean meal, containing more NDF, ADF, and EE [28]. In our study, the cottonseed meal had lower GE and CP digestibility than the SBM group. The study that found that in situ evaluation of DDGS partially replaced the soybean meal (diet without DDG or containing 150, 300, or 450 g/kg of inclusion) suggested that the inclusion of DDGS in finishing diets linearly reduced the ruminal degradability and the digestibility of DM and OM [29]. Therefore, though the different protein sources did not affect the body weight and ADG in this experiment period, the nutrient digestibility was higher in the SBM group, and it might impact the growth performance of lambs if the feeding period were longer. Meanwhile, nutrient intake can be affected by the NDF levels, the reticulorumen-filling effect, and the energy content in the diet; therefore, high NDF content may reduce the digestibility of other nutrients [30]. Animals fed diets with higher amounts of neutral detergent-insoluble fiber and/or larger particle sizes will spend greater time on chewing activities (feeding and rumination) [31]. Though the diet composition was similar among the treatments, the feed characteristics can alter the ingestive behavior parameters due to the diet interactions [32]. Compared to other protein sources, soybean meal has higher feed efficiency and apparent digestibility of DM, GE, and CP; however, the clear pathway needs further study. Though used in previous studies [33,34], the 4-day digestibility period may have limited the correct assessment of nutrients and energy digestibility.

### 4.2. Serum Parameters

The higher levels of ALT and AST serum concentrations in the COM group may be associated with the presence of gossypol in cottonseed meal [35]. Although not determined in the present study, free gossypol can affect the liver, causing increased serum liver enzymes such as ALT and AST [36]. During gossypol intoxication, liver cells are damaged and ALT and AST are released into the blood as hepatic cells are destroyed [37]. Free gossypol is toxic to animals when its accumulation reaches a high level as it can bind to proteins containing free amino sites and inhibit their normal metabolic pathways [38]. Serum concentrations of GLU, BUN, TP, and ALB are generally considered to be indicators of protein synthesis and metabolism related to the growth performance of ruminants [39]. The TP is significantly lower in the PEM group and supported by low albumin, indicating that protein synthesis and absorption was lower in this group [40]. Our study results illustrate little difference in the immune function but greater differences in protein synthesis and absorption among the different protein sources.

### 4.3. Rumen Fermentation and Bacterial Community

The MCP in the rumen is an essential indicator for the synthesis of microbial protein [41] from preformed amino acids and ammonia-N [42]. The minimum concentration of ammonia-N required for the maintenance of optimum microbial activity in the rumen is 5 mg/dL [43], and the BUN concentration indicates that the ruminal protein synthesis is balanced. The increase in CP degradability contributed to an accumulation of ruminal ammonia-N [44]. Therefore, our results illustrated that the increase in CP degradability contributed to an accumulation of ammonia-N in the rumen in the SBM group. In our study, the total replacement of soybean meal by other protein sources did not affect MCP synthesis, except for DDGS. The synchronism between the rumen availability of fermentable energy and degradable nitrogen is crucial for efficiency of MCP synthesis [45]. Thus, the lower MCP synthesis in the DDGS group might be explained by the lower availability of starch limiting the fermentable energy available for rumen microorganisms or its synchronism with rumen-degradable nitrogen. Previous studies indicated that compared to the soybean meal, rumen nitrogen and protein synthesis efficiency were similar in peanut meal [46] and cottonseed meal [47], possibly because of the different physiological statuses and animals (dairy cows and lambs). Additionally, in our study, the bacterial composition was not affected, indicating that the different sources of protein did not influence the microbial community and rumen fermentation. There is no significant difference in microbial communities, but the Anosim is 0.058, indicating a trend of changing the composition of rumen microbiota in different protein sources. Similarly to bacterial communities, rumen fermentation parameters were not affected by total replacement of soybean meal with the studied protein sources, except for lower ammonia-N content. Previous studies indicate that the Firmicutes-to-Bacteroidetes ratio is an important index of the health of microbiota [17] and VFA production [48]. The increase in the Firmicutes-to-Bacteroidetes ratio may be an indicator of a disorder in the microbial community [49], though the ratio was not significant among the five protein sources. However, a limitation of the data obtained on rumen fermentation and the bacterial community is that we only collected 50 mL of rumen fluid on the last day of the trial; therefore, we may have not captured all the changes that occurred during the experimental period.

## 5. Conclusions

Based on the above, the total replacement of soybean meal by other protein sources (cottonseed meal, peanut meal, rapeseed meal, or DDGS) in the diet of growing lambs does not impact the body weight and average daily gain, nor the immune function and rumen bacterial community. However, compared to soybean meal, other protein sources have lower rumen protein degradation to supply ammonia-N to the rumen and have lower apparent digestibility in GE and CP, which may increase the ADFI and decrease feed efficiency in growing lambs. Further studies are needed to assess the effects of soybean meal replacement (cottonseed meal, peanut meal, rapeseed meal, or DDGS) in long-term feeding trials.

## Figures and Tables

**Table 1:** Proportion of ingredients and chemical composition of different protein sources diet.

Ingredients, % DM	Dietary Treatment				
	Soybean Meal	Cottonseed Meal	Peanut Meal	Rapeseed Meal	DDGS ^1^
Peanut vine	22.50	22.50	23.00	17.50	17.80
Corn	55.80	55.50	56.00	54.50	39.00
Soybean meal	16.70				
Cottonseed cake		17.00			
Peanut cake			16.00		
Rapeseed cake				23.00	
DDGSs					37.70
NaHCO_3_	1.00	1.00	1.00	1.00	1.00
Premix ^2^	4.00	4.00	4.00	4.00	4.00
Total	100.00	100.00	100.00	100.00	100.00
Diet Composition					
Dry matter	89.57	89.20	89.93	89.02	90.09
Metabolizable energy ^3^, MJ kg^−1^ DM	12.10	12.07	12.00	12.02	12.19
Starch content	35.01	34.50	35.83	34.50	25.65
Crude protein	15.14	15.32	15.16	15.16	15.43
Neutral detergent fiber	40.16	40.35	39.61	37.63	40.50
Acid detergent fiber	17.65	19.06	21.49	18.57	20.29
Ether extract	3.07	2.98	3.33	3.26	2.93
Ash	8.59	8.95	8.84	9.01	8.89
Calcium	0.85	0.83	0.84	0.91	0.91
Phosphorus	0.31	0.31	0.30	0.33	0.38

^1^ DDGS: distillers’ dried grains with solubles. ^2^ The premix provided the following in kg of diet DM: vitamin A 17, 456 IU; vitamin D3, 3740 IU; vitamin E, 50 mg; Fe, 98.70 mg; Cu, 15.94 mg; Zn, 72.90 mg; Mn, 57.40 mg; Se, 0.33 mg; I, 1.30 mg; Co, 0.39 mg; NaCl, 3 g. ^3^ Metabolizable energy values calculated as digestible energy × 0.82.

**Table 2:** Effects of different protein source diets on growth performance and apparent nutrient digestibility of growing lambs.

Items ^1^	Groups						
	Soybean Meal	Cottonseed Meal	Peanut Meal	Rapeseed Meal	DDGS	SEM	*p*-Value
Growth performance							
IW, kg	38.6	37.3	39.8	39.3	38.62	0.403	0.325
FW, kg	51.4	50.4	52.3	53.1	51.54	0.548	0.599
ADG, g/d	246.4	252.1	248.9	266.9	251.40	5.829	0.687
ADFI, kg/d	1.46 ^b^	1.64 ^a^	1.63 ^a^	1.69 ^a^	1.64 ^a^	0.012	<0.001
Feed efficiency ^2^	5.94 ^a^	6.46 ^b^	6.80 ^c^	6.32 ^b^	6.51 ^b^	0.045	<0.001
Apparent nutrient digestibility, %							
DM	83.4 ^a^	82.6 ^a^	68.5 ^c^	83.4 ^a^	77.9 ^b^	0.513	<0.001
GE	75.4 ^a^	71.2^c^	60.7 ^d^	72.6 ^b^	71.1 ^c^	0.697	<0.001
CP	84.6 ^a^	80.3^c^	74.2 ^d^	83.0 ^b^	79.4 ^c^	0.440	<0.001
EE	82.8	80.6	85.0	81.6	80.2	1.307	0.203
NDF	58.9	62.4	59.1	63.4	60.2	1.243	0.284
ADF	23.1	29.6	28.2	33.3	24.0	1.856	0.263

^a, b, c, d^ Values within a row with different superscripts differ significantly at *p* < 0.05. ^1^ DDGS: distillers’ dried grains with solubles; IW: initial live weight; FW: final live weight; ADG: average daily gain in live weight; ADFI: average daily feed intake; DM: dry matter; GE: gross energy; CP: crude protein; EE: ether extract; NDF: neutral detergent fiber; ADF: acid detergent fiber. ^2^ Feed efficiency = ADFI/ADG.

**Table 3:** Effects of different protein sources diets on serum parameters of growing lambs.

Items	Groups						
	Soybean Meal	Cottonseed Meal	Peanut Meal	Rapeseed Meal	DDGS	SEM	*p*-Value
Immunoglobulin, mg/mL							
Immunoglobulin A	1.52	1.81	1.66	1.72	1.51	0.045	0.176
Immunoglobulin G	13.43 ^a, b^	13.37 ^a, b^	14.20 ^a^	10.28 ^c^	12.22 ^b^	0.391	<0.001
Immunoglobulin M	1.76 ^a^	1.70 ^b^	1.73 ^a, b^	1.72 ^a, b^	1.75 ^a^	0.007	0.016
Metabolism							
Glucose, mmol/L	5.02	5.38	4.71	4.99	4.94	0.135	0.670
Blood urea nitrogen, mmol/L	8.74 ^a^	7.41 ^a^	6.05 ^b^	5.37 ^b^	8.21 ^a^	0.300	<0.001
Total protein, g/L	83.00 ^a^	80.42 ^a^	80.73 ^a^	69.02 ^b^	78.98 ^a^	1.122	<0.001
Albumin, g/L	27.74 ^a^	28.69 ^a^	28.00 ^a^	24.37 ^b^	26.80 ^a, b^	0.500	0.033
Globulin, g/L	55.31 ^a^	48.35 ^a, b^	52.41 ^a, b^	44.79 ^b^	49.10 ^a, b^	1.208	0.050
Total cholesterol, mmol/L	1.45	1.55	1.48	1.55	1.76	0.058	0.532
Aspartate aminotransferase, U/L	6.95 ^a, b^	8.38 ^a^	6.20 ^b^	7.04 ^a, b^	7.57 ^a, b^	0.300	0.188
Alanine aminotransferase, U/L	1.87 ^b^	3.40 ^a^	1.95 ^b^	1.91 ^b^	1.73 ^b^	1.576	<0.001

^a, b^ Values within a row with different superscripts differ significantly at *p* < 0.05.

**Table 4:** Effects of different protein sources diets on rumen fermentation parameters of growing lambs.

Items ^1^, %	Groups						
	Soybean Meal	Cottonseed Meal	Peanut Meal	Rapeseed Meal	DDGS	SEM	*p*-Value
pH	6.18	6.27	6.35	6.20	6.13	0.685	0.303
NH_3_-N, mg/dL	20.68 ^a^	12.55 ^b^	11.52 ^b^	11.18 ^b^	11.01 ^b^	0.712	<0.001
MCP, mg/dL	9.09 ^a^	8.10 ^a, b^	8.79 ^a, b^	8.42 ^a, b^	7.75 ^b^	0.161	0.049
Acetate, mmol/L	31.33	34.50	27.26	32.57	28.76	1.957	0.803
Propionate, mmol/L	32.95	27.92	21.92	34.38	28.58	1.852	0.234
Butyrate, mmol/L	10.31	10.29	8.96	10.38	8.03	0.858	0.8993
Valerate, mmol/L	1.61	2.17	2.05	1.89	2.03	1.579	0.850
Isobutyrate	0.69	0.13	0.27	0.09	0.03	0.103	0.267
Isovalerate	0.85	0.54	0.88	0.40	0.25	0.107	0.254
Total volatile fatty acids, mmol/L	77.74	75.55	61.34	79.70	67.68	3.471	0.438
Acetate/Propionate	1.38	1.32	1.31	0.95	1.03	0.124	0.778

^a, b^ Values within a row with different superscripts differ significantly at *p* < 0.05. ^1^ MCP: microbial crude protein; DDGS: distillers’ dried grains with solubles.

**Table 5:** Effects of different protein source diets on rumen bacterial community of growing lambs at the phylum level (relative abundance > 1%).

Items ^1^	Groups						
	Soybean Meal	Cottonseed Meal	Peanut Meal	Rapeseed Meal	DDGS	SEM	*p*-Value
Firmicutes	45.14	54.04	56.21	43.24	47.32	1.939	0.138
Bacteroidota	38.35	30.74	30.38	43.18	32.21	1.692	0.319
Proteobacteria	10.55	10.09	10.17	12.34	12.89	1.024	0.764
Actinobacteriota	4.34	6.45	5.66	4.76	5.44	0.787	0.285
Firmicutes/Bacteroidota	1.22	2.27	1.98	1.17	1.56	0.167	0.151

^1^ DDGS: distillers’ dried grains with solubles.

**Table 6:** Effects of different protein source diets on rumen bacterial community of growing lambs at the genus level (relative abundance > 1%).

Items ^1^	Groups						
	Soybean Meal	Cottonseed Meal	Peanut Meal	Rapeseed Meal	DDGS	SEM	*p*-Value
*Prevotella 7*	26.98	28.06	20.25	21.93	20.66	2.020	0.713
*Dialister*	8.31	7.12	5.97	8.33	5.80	0.627	0.569
*Lachnospiracea NK3A20 group*	5.76	11.56	7.81	6.09	9.05	0.889	0.242
*Unclassified Selenomonadaceae*	5.24	4.63	2.78	6.45	6.87	0.679	0.410
*Prevotella*	4.00	6.98	5.48	3.48	6.96	1.098	0.829
*Succinivibrionaceae UCG 001*	2.22	5.68	2.10	3.11	5.13	0.700	0.391
*Rikenellaceae RC9 gut group*	3.97	2.33	2.99	3.92	2.20	0.392	0.472
*Olsenella*	1.33	3.53	3.71	3.27	3.46	0.372	0.177
*Unclassified Lachnospiraceae*	1.72	3.20	1.66	1.44	1.35	0.282	0.259
*Succinivibrio*	1.19	0.51	2.03	0.13	0.49	0.318	0.388

^1^ DDGS: distillers’ dried grains with solubles.

## Data Availability

The datasets analyzed in this study can be found in the Genome Sequence Archive repository (http://gsa.big.ac.cn). Please see accession number PRJCA024076 for more details.
